# Association between anemia and household water source or sanitation in preschool children: the Biomarkers Reflecting Inflammation and Nutritional Determinants of Anemia (BRINDA) project

**DOI:** 10.1093/ajcn/nqaa148

**Published:** 2020-08-04

**Authors:** Emma X Yu, O Yaw Addo, Anne M Williams, Reina Engle-Stone, Jiangda Ou, Weixing Huang, Junjie Guo, Parminder S Suchdev, Melissa F Young

**Affiliations:** Hubert Department of Global Health, Rollins School of Public Health, Emory University, Atlanta, GA, USA; Hubert Department of Global Health, Rollins School of Public Health, Emory University, Atlanta, GA, USA; Nutrition Branch, Centers for Disease Control and Prevention, Atlanta, GA, USA; McKing Consultation Corporation, Atlanta, GA, USA; Hubert Department of Global Health, Rollins School of Public Health, Emory University, Atlanta, GA, USA; Nutrition Branch, Centers for Disease Control and Prevention, Atlanta, GA, USA; McKing Consultation Corporation, Atlanta, GA, USA; Department of Nutrition, University of California, Davis, CA, USA; Hubert Department of Global Health, Rollins School of Public Health, Emory University, Atlanta, GA, USA; Hubert Department of Global Health, Rollins School of Public Health, Emory University, Atlanta, GA, USA; Hubert Department of Global Health, Rollins School of Public Health, Emory University, Atlanta, GA, USA; Hubert Department of Global Health, Rollins School of Public Health, Emory University, Atlanta, GA, USA; Nutrition Branch, Centers for Disease Control and Prevention, Atlanta, GA, USA; Department of Pediatrics, Emory University, Atlanta, GA, USA; Hubert Department of Global Health, Rollins School of Public Health, Emory University, Atlanta, GA, USA

**Keywords:** anemia, water, sanitation, complex survey, preschool children, BRINDA

## Abstract

**Background:**

The associations between anemia and household water source and sanitation remain unclear.

**Objectives:**

We aimed to assess the associations between anemia and household water source or sanitation in preschool children (PSC; age 6–59 mo) using population-based surveys from the Biomarkers Reflecting Inflammation and Nutritional Determinants of Anemia (BRINDA) project.

**Methods:**

We analyzed national and subnational data from 21 surveys, representing 19 countries (*n* = 35,963). Observations with hemoglobin (Hb) and ≥1 variable reflecting household water source or sanitation were included. Anemia was defined as an altitude-adjusted Hb concentration <110 g/L. Household water source and sanitation variables were dichotomized as “improved” or “unimproved.” Poisson regressions with robust variance estimates were conducted for each survey, adjusting for child sex, age, household socioeconomic status, maternal education, and type of residence.

**Results:**

Access to an improved water source and improved sanitation ranged from 29.9% (Burkina Faso) to 98.4% (Bangladesh, 2012), and from 0.2% (Kenya, 2007) to 97.4% (Philippines), respectively. Prevalence of anemia ranged from 20.1% (Nicaragua) to 83.5% (Bangladesh, 2010). Seven surveys showed negative associations between anemia and improved sanitation. Three surveys showed association between anemia and improved water, with mixed directions. Meta-analyses suggested a protective association between improved household sanitation and anemia [adjusted prevalence ratio (aPR) = 0.88; 95% CI: 0.79, 0.98], and no association between improved household water and anemia (aPR = 1.00; 95% CI: 0.91, 1.10). There was heterogeneity across surveys for sanitation (*P *< 0.01; *I*^2^ = 66.3%) and water (*P *< 0.01; *I*^2^ = 55.8%).

**Conclusions:**

Although improved household sanitation was associated with reduced anemia prevalence in PSC in some surveys, this association was not consistent. Access to an improved water source in general had no association with anemia across surveys. Additional research could help clarify the heterogeneity between these conditions across countries to inform anemia reduction programs.

## Introduction

Anemia remains a worldwide public health problem and has substantial adverse health consequences (1), as well as economic impact (1,2). Compared with other vulnerable groups, preschool children (PSC; 6–59 mo of age) continue to have the highest prevalence of anemia (3). In 2016, the prevalence of anemia was estimated to be 41.7% in PSC (4), impacting 280 million children worldwide (5).

The etiology of anemia in PSC is multifaceted and context specific (6,7), involving complex interplay between nutritional status, infections, environmental exposures, and other factors (1). Nutrition interventions alone without environment interventions might not be sufficient to control anemia in specific settings (8). Improved water and sanitation can influence anemia by reducing diarrheal diseases, environmental enteric dysfunction, and parasitic infections (9–11). However, despite the efforts to increase access to improved water, sanitation, and hygiene (WASH) worldwide, an estimated 55% of the global population still did not have access to safely managed sanitation in 2017 (12). Meanwhile, an estimated 29% of the global population did not use a safely managed drinking water service that was free from contamination, located on premises, and available when needed (12).

The existing evidence on the relation between anemia and household water source or sanitation from randomized clinical trials (13–15) or observational studies (16–19) is mixed. For example, in Kenya, a WASH + Nutrition intervention resulted in a nonsignificant 8.9% lower anemia prevalence compared with a Nutrition-only intervention, whereas there was no added value of WASH + Nutrition over Nutrition alone in its companion trial in Bangladesh (13). These mixed results could reflect variation in the context in which the studies were conducted. Therefore we leveraged the Biomarkers Reflecting Inflammation and Nutritional Determinants of Anemia (BRINDA) database to examine the associations between anemia and household water source or sanitation in a variety of settings.

## Methods

### Data sources

**FIGURE 1:**
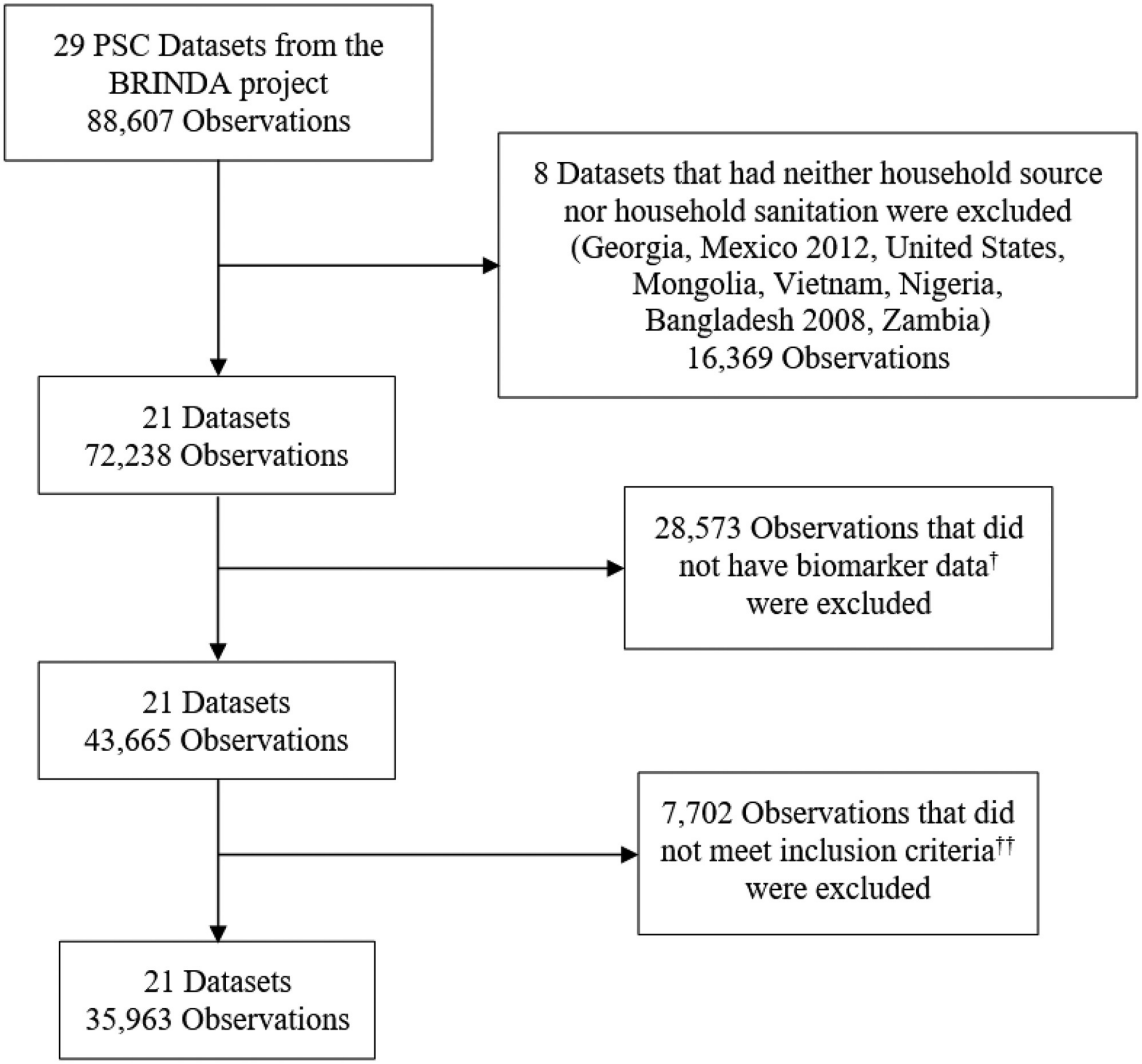
Sample size for the analysis of association between anemia and household water source or sanitation in preschool children: the Biomarkers Reflecting Inflammation and Nutritional Determinants of Anemia (BRINDA) project. ^†^Biomarker data meant having at least 1 biomarker (hemoglobin, ferritin, transferrin receptor, retinol, retinol-binding protein, zinc, vitamin B12, folate or red blood cell folate). ^††^Inclusion criteria: having biologically plausible hemoglobin value adjusted for altitude and ≥1 variable reflecting household water source or sanitation.

We analyzed nationally and subnationally representative data from the BRINDA project. The references for the original surveys are available on the BRINDA website (www.BRINDA-nutrition.org) (20). The inclusion and exclusion criteria and data management for the BRINDA project have been described in detail elsewhere (20). In brief, we included individuals with biologically plausible hemoglobin (Hb) concentrations (40–180 g/L) (21) and ≥1 variable reflecting household water source or sanitation. Eight of 29 surveys had neither household water source nor household sanitation information and were thus excluded, specifically Georgia, Mexico (2012), United States, Mongolia, Vietnam, Nigeria, Bangladesh (2008), and Zambia (**Figure 1**). Two surveys [Mexico (2006) and Côte d'Ivoire] that measured household sanitation but not household water source were included.

### Blood analysis

Venous or capillary blood was collected from children and whole blood was assessed for Hb using a variety of analyzers, although Hemocue 201+ was the most common (**Supplemental Table 1)**. The sandwich ELISA was the dominant method for assessment of C-reactive protein (CRP) and α-1-acid glycoprotein (AGP) in serum or plasma; laboratory methods for the biomarkers in each survey are given in Supplemental Table 1.

### Case definitions

The primary outcome was anemia, which was defined as Hb concentration <110 g/L (22). Hb concentrations were adjusted for altitude when this information was available, specifically Afghanistan, Azerbaijan, Colombia, Ecuador, Laos, Malawi, Mexico (2006), Papua New Guinea, and Rwanda (Supplemental Table 1). The primary exposure was household water source or sanitation. For consistency across surveys and due to insufficient sample size within some of the water source or sanitation levels, we dichotomized water source and sanitation as either improved or unimproved household drinking water source, and improved or unimproved sanitation, respectively. The dichotomization was adapted from the WHO/UNICEF Joint Monitoring Programme classification (23). Improved water services were those that had the potential to deliver safe water, including piped supplies (tap water in the dwelling, yard or plot, public standposts), and nonpiped supplies (boreholes/tubewells, protected wells and springs, rainwater, packaged water, and delivered water) (23). Improved sanitation services were those designed to hygienically separate excreta from human contact, including networked sanitation (flush and pour-flush toilets connected to sewers), and on-site sanitation (flush and pour-flush toilets or latrines connected to septic tanks or pits, ventilated improved pit latrines, pit latrines with slabs, and composting toilets) (23).

Covariates included child age measured in months, household socioeconomic status (SES), child sex, maternal education level (no education, primary education, secondary education, or university), and type of residence (rural or urban), defined a priori. Household SES was a 3-level ordinal variable created from asset quintiles or country income variables. Due to insufficient sample size within some of the asset levels, it was necessary to collapse across levels within each survey. Specifically, the survey-specific first and second quintiles/categories were collapsed as “low SES,” the third and fourth quintiles/categories were collapsed as “medium SES,” and the fifth quintile/category was converted as “high SES.” Inflammation was a binary variable, defined as CRP concentration >5 mg/L or AGP concentration >1 g/L (24). We have summarized the laboratory methods for Hb, CRP, and AGP in Supplemental Table 1.

### Statistical analysis

We examined household sociodemographic characteristics, improved water source or sanitation, and prevalence of inflammation and anemia as percentages using PROC SURVEYFREQ (SAS Institute Inc). Medians (minimum to maximum) for continuous age were determined using PROC SURVEYMEANS (SAS Institute Inc). We used Poisson regressions with log link function and robust variance estimation to calculate associations [prevalence ratio (PR); 95% CI] between anemia and household water source or sanitation. We previously tested for interaction between water and sanitation in predicting anemia and found no significance at the significance level of 0.10 for all surveys except for Bangladesh (2010 and 2012) (data not shown). We thus conducted modeling using water and sanitation as 2 independent predictors.

Crude models were first examined and then adjusted for sex, child age, household SES, maternal education, and/or type of residence. We further adjusted for malaria for the 6 surveys with this variable as a sensitivity analysis. Malaria was classified using plasma histidine-rich protein 2 in Cameroon, microscopy in Côte d'Ivoire and Kenya, and a rapid diagnostic test in Liberia and Malawi. A second adjusted model that included the covariates above plus inflammation was built to examine whether inflammation mediated the potential relation between anemia and household water source or sanitation. Finally, survey-specific PR estimates were meta-analyzed with the use of a random-effects model (25). We assessed the heterogeneity with the use of *P* value from the Cochran Q test and the *I*^2^ statistic (26). Statistically significant heterogeneity was indicated by a *P* value < 0.10 from Cochran Q (27). If significant, the *I*^2^ statistic was further interpreted as: minimum heterogeneity (0–30%); moderate heterogeneity (30–60%); substantial heterogeneity (60–90%); and considerable heterogeneity (90–100%), depending on magnitude and direction of effects. This categorization was adapted from the Cochrane handbook for systematic reviews of interventions (26).

All statistical tests were 2-sided and used a significance level of *P* < 0.05. Analyses were performed in SAS version 9.4 software (SAS Institute Inc), taking into account complex survey design (i.e., sampling weight, cluster, and stratum, which were computed based on the sampling design of each survey and recoded from the raw data). We performed the meta-analyses of PRs and created forest plots with the use of the metafor package in R 3.5.0 software (28).

## Results

We included a sample size of 35,963 PSC from 21 surveys, representing 19 countries (Figure 1). The age range of participants differed by survey from 6–11 mo in Bangladesh (2010), 12–59 mo in Cameroon and Mexico, 6–23 mo in the Philippines, 35–59 mo in Burkina Faso, 6–35 mo in Kenya (2010 and 2011) and Liberia, to 6–59 mo in other surveys (**Table 1**). There was also variation in SES and type of residence across the 21 surveys (Table 1). Only 24% of participants in Mexico were from rural areas whereas all the participants in the 2 Kenyan subnational surveys had rural residence (Table 1).

**TABLE 1:** Sociodemographic characteristics of preschool children by survey, BRINDA project^1^

Country (year)	Total *n*	*n*1^2^	*n*2^3^	Male, % (95% CI)	Median age, mo (min–max)	Low SES,^4^ % (95% CI)	The child's mother had no education, %	Rural, % (95% CI)
Afghanistan (2013)	19,896	876	809	56.1 (51.3, 60.8)	26.4 (6–58)	5.5 (3.1, 7.9)	N/A	N/A
Azerbaijan (2013)	1404	1090	1088	55.4 (51.4, 59.5)	36.1 (6–59)	32.8 (28.1, 37.4)	N/A	54.7 (47.6, 61.9)
Bangladesh (2010)	1561	1499	1496	49.3 (47.1, 51.5)	7.7 (6–11)	N/A	N/A	N/A
Bangladesh (2012)	1108	946	607	44.8 (36.1, 53.4)	37.4 (6–59)	53.6 (43.6, 63.6)	15.7 (10.5, 20.9)	74.8 (67.9, 81.7)
Burkina Faso (2010)	482	152	85	44.6 (28.6, 60.7)	49.0 (35–59)	23.7 (7.2, 40.1)	N/A	N/A
Cambodia (2014)	874	793	536	55.7 (51.7, 59.7)	32.4 (6–59)	42.3 (33.0, 51.6)	14.3 (8.6, 19.9)	85.8 (79.5, 92.1)
Cameroon (2009)	853	847	794	50.2 (46.9, 53.5)	29.8 (12–59)	44.4 (37.1, 51.7)	27.7 (24.7, 30.8)	41.4 (31.5, 51.3)
Colombia (2010)	7753	7642	1637	52.4 (49.5, 55.2)	30.2 (6–59)	53.4 (50.8, 56.0)	N/A	32.4 (30.9, 34.0)
Côte d'Ivoire (2007)	864	834	552	51.7 (47.1, 56.3)	29.8 (6–59)	19.6 (15.3, 23.9)	53.3 (46.0, 60.5)	30.4 (24.2, 36.6)
Ecuador (2012)	10,202	2020	2018	49.4 (45.3, 53.5)	29.2 (6–59)	49.1 (43.5, 54.6)	1.2 (0.7, 1.7)	34.8 (22.8, 46.8)
Kenya (2007)	1056	1043	991	51.8 (49.0, 54.6)	19.3 (6–35)	41.1 (36.0, 46.2)	2.5 (1.6, 3.4)	100
Kenya (2010)	896	860	832	50.6 (46.9, 54.3)	22.3 (6–35)	39.7 (34.3, 45.1)	1.6 (0.6, 2.6)	100
Laos (2006)	514	497	494	50.1 (45.6, 54.6)	33.1 (6–59)	60.8 (50.4, 71.3)	35.2 (26.7, 43.7)	85.7 (77.0, 94.4)
Liberia (2011)	1476	1457	1453	50.4 (47.6, 53.3)	19.0 (6–35)	36.3 (30.1, 42.5)	N/A	63.1 (59.6, 66.6)
Malawi (2016)	1233	1189	1170	50.1 (47.2, 53.0)	32.0 (6–59)	49.8 (44.0, 55.6)	11.1 (7.0, 15.3)	90.4 (80.3, 100.0)
Mexico (2006)	6618	6617	6270	50.4 (48.4, 52.3)	38.1 (12–59)	46.6 (44.2, 49.1)	N/A	24.1 (21.8, 26.4)
Nicaragua (2005)	1424	1424	1420	49.8 (46.5, 53.1)	34.3 (6–59)	N/A	17.0 (13.0, 21.0)	43.7 (31.4, 55.9)
Pakistan (2011)	10,689	10,608	10,447	52.3 (51.2, 53.5)	24.4 (6–59)	42.5 (40.3, 44.8)	57.6 (55.7, 59.5)	69.8 (67.2, 72.5)
PNG (2005)	934	911	907	55.0 (51.8, 58.2)	30.4 (6–59)	40.1 (29.0, 51.3)	N/A	80.6 (71.4, 89.8)
Philippines (2011)	1784	1784	1782	49.9 (46.9, 53.0)	15.3 (6–23)	84.4 (81.3, 87.6)	6.0 (4.3, 7.7)	90.8 (90.2, 91.5)
Rwanda (2010)	617	576	575	47.6 (43.6, 51.7)	34.3 (6–59)	N/A	N/A	N/A

1 We examined household sociodemographic characteristics as percentages using PROC SURVEYFREQ (SAS Institute Inc). Medians (minimum to maximum) for continuous age were determined using PROC SURVEYMEANS (SAS Institute Inc). BRINDA, Biomarkers Reflecting Inflammation and Nutritional Determinants of Anemia; max, maximum; min, minimum; N/A, not available; PNG, Papua New Guinea; SES, socioeconomic status.

2 *n*1 was the biomarker sample size, obtained after applying an inclusion criterion of observations with ≥1 biomarker (hemoglobin, ferritin, transferrin receptor, retinol, retinol-binding protein, zinc, vitamin B-12, folate, or red blood cell folate).

3 *n*2 was the analytic sample size, obtained after applying an inclusion criterion of observations with a biologically plausible hemoglobin concentration (40–180 g/L) and a variable reflecting household water source or sanitation.

4 SES was a 3-level ordinal variable created from survey-specific asset quintiles or country income variables. Specifically, the first and second quintiles/categories were collapsed as “low SES,” the third and fourth quintiles/categories were collapsed as “medium SES,” and the fifth quintile/category was converted as “high SES.”

Children with biomarker data excluded because of missing or implausible Hb values or missing water source or sanitation variables (*n* = 7702) did not differ from those who were included (*n* = 35,963) with regard to sex except that children excluded in Pakistan were more likely to be females than were children included (data not shown). Excluded children were older in Cambodia, more likely to have low SES in 4 surveys, and more likely to reside in rural areas in 4 surveys than were children included (data not shown). In addition, mothers of excluded children were more likely to have no education in Côte d'Ivoire, Kenya 2007, and Pakistan (data not shown).

The percentage of participants with access to an improved household water source ranged from 29.9% (95% CI: 10.7%, 49.0%) in Burkina Faso to 98.4% (95% CI: 96.5%, 100%) in Bangladesh (2012) whereas the percentage of participants with access to improved household sanitation ranged from 0.2% (95% CI: 0.0%, 0.5%) in Kenya (2007) to 97.4% (95% CI: 95.7%, 99.1%) in the Philippines (**Table 2**). Anemia prevalence in PSC ranged from 20.1% (95% CI: 15.6%, 24.7%) in Nicaragua to 83.5% (95% CI: 81.1%, 85.9%) in Bangladesh (2010) (Table 2). Inflammation prevalence ranged from 10.8% (95% CI: 8.5%, 13.1%) in Mexico to 94.3% (95% CI: 88.6%, 100%) in Burkina Faso (Table 2).

**TABLE 2:** Prevalence of an improved household water source, household sanitation, inflammation, and anemia in preschool children by survey, BRINDA project^1^

Country (year)	Improved household water	Improved household sanitation	Inflammation	Anemia
Afghanistan (2013)	81.2 (74.8, 87.6)	63.1 (55.0, 71.3)	24.1 (19.2, 29.0)	43.8 (36.9, 50.8)
Azerbaijan (2013)	79.9 (74.5, 85.2)	93.1 (89.8, 96.4)	30.9 (27.2, 34.6)	24.5 (21.1, 28.0)
Bangladesh (2010)	98.1 (94.4, 100.0)	26.3 (16.8, 35.9)	35.7 (32.1, 39.3)	83.5 (81.1, 85.9)
Bangladesh (2012)	98.4 (96.5, 100.0)	66.2 (52.5, 79.8)	29.3 (23.1, 35.6)	33.1 (25.7, 40.5)
Burkina Faso (2010)	29.9 (10.7, 49.0)	13.6 (0.0, 29.2)	94.3 (88.6, 100.0)	72.0 (64.1, 79.8)
Cambodia (2014)	57.2 (47.3, 67.1)	52.5 (43.3, 61.7)	38.5 (30.8, 46.3)	55.7 (50.6, 60.7)
Cameroon (2009)	72.3 (66.8, 77.8)	62.7 (56.9, 68.4)	48.2 (42.9, 53.5)	55.0 (50.1, 59.9)
Colombia (2010)	87.7 (85.6, 89.8)	96.5 (95.2, 97.8)	18.2 (14.9, 21.5)	25.5 (22.9, 28.2)
Côte d'Ivoire (2007)	N/A	89.2 (85.0, 93.3)	61.9 (57.1, 66.6)	71.5 (67.5, 75.5)
Ecuador (2012)	76.5 (71.3, 81.7)	94.6 (93.2, 96.0)	12.5 (10.1, 14.9)	24.7 (20.9, 28.5)
Kenya (2007)	50.9 (40.8, 60.9)	0.2 (0.0, 0.5)	65.3 (61.1, 69.6)	65.8 (62.0, 69.6)
Kenya (2010)	54.3 (45.0, 63.6)	1.1 (0.2, 2.1)	61.9 (57.3, 66.5)	72.0 (68.5, 75.5)
Laos (2006)	44.1 (32.4, 55.7)	87.1 (77.7, 96.5)	43.8 (36.3, 51.3)	40.7 (31.5, 49.9)
Liberia (2011)	83.2 (76.2, 90.1)	53.0 (45.1, 60.9)	59.0 (55.5, 62.6)	59.3 (55.5, 63.2)
Malawi (2016)	83.5 (76.8, 90.1)	83.4 (79.2, 87.5)	56.8 (51.1, 62.5)	31.4 (27.7, 35.2)
Mexico (2006)	N/A	88.8 (87.1, 90.4)	10.8 (8.5, 13.1)	24.8 (23.0, 26.6)
Nicaragua (2005)	89.9 (85.3, 94.4)	29.7 (22.5, 36.9)	24.0 (20.5, 27.5)	20.1 (15.6, 24.7)
Pakistan (2011)	94.5 (93.5, 95.5)	94.9 (94.2, 95.7)	35.5 (34.0, 36.9)	62.9 (61.6, 64.1)
Papua New Guinea (2005)	66.0 (56.5, 75.5)	9.9 (3.7, 16.1)	57.0 (52.5, 61.5)	47.8 (42.4, 53.3)
Philippines (2011)	44.8 (40.4, 49.2)	97.4 (95.7, 99.1)	25.7 (22.2, 29.2)	41.7 (37.7, 45.6)
Rwanda (2010)	58.4 (50.8, 66.1)	3.5 (0.8, 6.1)	28.6 (23.7, 33.4)	27.1 (22.5, 31.7)

1 All values are proportions (95% CIs). Inflammation was defined as having a C-reactive protein concentration >5 mg/L or α-1-acid glycoprotein concentration >1 g/L. Anemia was defined as having an altitude-adjusted hemoglobin concentration <110 g/L, except for Bangladesh (2010 and 2012), Cambodia, Nicaragua, Pakistan, Philippines, Burkina Faso, Cameroon, Côte d'Ivoire, Kenya (2007 and 2010), and Liberia, where altitude was not available. We examined household improved water source or sanitation, and prevalence of inflammation and anemia as percentages using PROC SURVEYFREQ (SAS Institute Inc). BRINDA, Biomarkers Reflecting Inflammation and Nutritional Determinants of Anemia; N/A, not available.

An improved household water source was negatively associated with anemia in Azerbaijan [crude prevalence ratio (cPR) = 0.75; 95% CI: 0.57, 0.98], Cameroon (cPR = 0.82; 95% CI: 0.70, 0.96), and Laos (cPR = 0.72; 95% CI: 0.52, 0.999) but positively associated with anemia in Afghanistan (cPR = 1.42; 95% CI: 1.03, 1.94) and the Philippines (cPR = 1.40; 95% CI: 1.18, 1.66) (**Table 3**). After adjusting for child sex, child age in months, household SES, maternal education, and/or type of residence, an improved household water source was negatively associated with anemia in Laos [adjusted prevalence ratio (aPR) = 0.67; 95% CI: 0.49, 0.90) but positively associated with anemia in Afghanistan (aPR = 1.38; 95% CI: 1.01, 1.89) and the Philippines (aPR = 1.32; 95% CI: 1.10, 1.58) (Table 3). We did not observe an association between anemia and household water source in other surveys (Table 3). Adding malaria to the model did not significantly change the direction or magnitude of the associations (**Supplemental Table 2**). After further adding inflammation in the model, the association between anemia and an improved household water source changed <10% in most surveys (Table 3). However, the association became nonsignificant in Afghanistan and significant in Liberia (Table 3).

**TABLE 3:** Associations between anemia and an improved household water source in preschool children by survey, BRINDA project^1^

Country (year)	*n*	Crude PR (95% CI)	*n*	Adjusted PR-1 (95% CI)^2^	n	Adjusted PR-2 (95% CI)^3^
Afghanistan (2013)	804	1.42 (1.03, 1.94)*	804	1.46 (1.09, 1.96)*	600	1.34 (0.96, 1.87)
Azerbaijan (2013)	1062	0.75 (0.57, 0.98)*	1060	0.82 (0.64, 1.06)	1023	0.83 (0.63, 1.10)
Bangladesh (2010)	1481	1.02 (0.94, 1.11)	1481	1.01 (0.93, 1.11)	1475	1.01 (0.93, 1.10)
Bangladesh (2012)	607	0.60 (0.24, 1.55)	607	0.47 (0.17, 1.27)	452	0.51 (0.16, 1.65)
Burkina Faso (2010)	85	1.14 (0.93, 1.41)	85	1.18 (0.92, 1.51)	68	1.24 (0.95, 1.63)
Cambodia (2014)	536	0.98 (0.82, 1.17)	485	1.01 (0.85, 1.20)	367	0.98 (0.79, 1.22)
Cameroon (2009)	777	0.82 (0.70, 0.96)*	770	0.95 (0.83, 1.10)	722	1.01 (0.88, 1.17)
Colombia (2010)	1607	0.97 (0.73, 1.31)	1607	1.01 (0.78, 1.31)	805	1.42 (0.67, 3.03)
Côte d'Ivoire (2007)	N/A	N/A	N/A	N/A	N/A	N/A
Ecuador (2012)	2018	0.92 (0.73, 1.16)	1996	1.14 (0.92, 1.41)	1996	1.14 (0.92, 1.41)
Kenya (2007)	989	1.06 (0.96, 1.16)	988	1.06 (0.96, 1.17)	848	1.06 (0.96, 1.18)
Kenya (2010)	832	0.95 (0.87, 1.04)	824	0.97 (0.88, 1.06)	814	1.03 (0.95, 1.12)
Laos (2006)	493	0.72 (0.52, 0.999)*	465	0.67 (0.49, 0.90)**	450	0.67 (0.49, 0.92)*
Liberia (2011)	1436	1.16 (0.96, 1.39)	1436	1.20 (0.99, 1.45)	1414	1.25 (1.04, 1.50)*
Malawi (2016)	1168	0.94 (0.73, 1.20)	1046	0.98 (0.73, 1.31)	973	1.14 (0.82, 1.58)
Mexico (2006)	N/A	N/A	N/A	N/A	N/A	N/A
Nicaragua (2005)	1406	0.74 (0.46, 1.21)	1406	1.13 (0.70, 1.83)	1406	1.14 (0.70, 1.86)
Pakistan (2011)	10,261	0.96 (0.88, 1.05)	10,167	0.96 (0.88, 1.05)	7251	0.96 (0.87, 1.06)
Papua New Guinea (2005)	842	0.82 (0.65, 1.05)	839	0.85 (0.66, 1.09)	807	0.86 (0.68, 1.10)
Philippines (2011)	1776	1.40 (1.18, 1.66)**	1724	1.32 (1.10, 1.58)**	1707	1.30 (1.09, 1.55)**
Rwanda (2010)	573	0.99 (0.74, 1.33)	570	0.95 (0.71, 1.27)	570	0.95 (0.72, 1.27)

1 Values are prevalence ratios (PRs) (95% CIs). Unimproved household water source is the reference group. Anemia was defined as having an altitude-adjusted hemoglobin concentration <110 g/L, except for Bangladesh (2010 and 2012), Cambodia, Nicaragua, Pakistan, Philippines, Burkina Faso, Cameroon, Côte d'Ivoire, Kenya (2007 and 2010), and Liberia, where altitude was not available. We used Poisson regressions with log link function and robust variance estimation to calculate associations (PR; 95% CI) between anemia and household water source or sanitation; ^*,**^significant association: *P < 0.05, **P < 0.01. AGP, α-1-acid glycoprotein; BRINDA, Biomarkers Reflecting Inflammation and Nutritional Determinants of Anemia; CRP, C-reactive protein; N/A, not available; SES, socioeconomic status.

2 Adjusted for child sex, child age in months (continuous), household SES, maternal education (no education, primary education, secondary education, or university), and/or type of residence (rural/urban), when available. SES was a 3-level ordinal variable created from asset quintiles or country income variables. Specifically, the first and second quintiles/categories were collapsed as “low SES,” the third and fourth quintiles/categories were collapsed as “medium SES,” and the fifth quintile/category was converted as “high SES.”

3 Adjusted for child sex, child age in months (continuous), household SES, maternal education (no education, primary education, secondary education, or university), and/or type of residence (rural/urban), when available. Further adjusted for inflammation (yes/no). Inflammation was defined as having a CRP concentration >5 mg/L or an AGP concentration >1 g/L.

With regard to the relations between anemia and improved household sanitation, improved household sanitation was associated with a lower prevalence of anemia in 10 of 20 surveys that could be estimated (**Table 4**). After adjusting for child sex, child age in months, household SES, maternal education, and/or type of residence, improved household sanitation was negatively associated with anemia in 7 of 20 surveys (Table 4). We did not observe an association between anemia and household sanitation in other surveys (Table 4). Adding malaria to the model did not significantly change the direction or magnitude of the associations (Supplemental Table 2). Adding inflammation in the model did not impact effect estimates in most surveys. However, the association between anemia and improved household sanitation became nonsignificant in Afghanistan and Azerbaijan, and became significant in Cambodia and Côte d'Ivoire. There was a 13.4% reduction in the estimate for Burkina Faso, whereas the change was <10% in other surveys (Table 4).

**TABLE 4:** Associations between anemia and improved household sanitation in preschool children by survey, BRINDA project^1^

Country (year)	*n*	Crude PR (95% CI)	*n*	Adjusted PR-1 (95% CI)^2^	*n*	Adjusted PR-2 (95% CI)^3^
Afghanistan (2013)	796	0.73 (0.58, 0.93)*	796	0.74 (0.56, 0.97)*	591	0.90 (0.66, 1.23)
Azerbaijan (2013)	1083	0.69 (0.49, 0.96)*	1081	0.70 (0.5, 0.97)*	1044	0.79 (0.58, 1.08)
Bangladesh (2010)	1416	0.96 (0.91, 1.01)	1416	0.96 (0.90, 1.01)	1410	0.96 (0.91, 1.02)
Bangladesh (2012)	595	0.79 (0.54, 1.17)	595	0.81 (0.55, 1.21)	443	0.69 (0.45, 1.06)
Burkina Faso (2010)	85	0.68 (0.48, 0.98)*	85	0.67 (0.45, 0.98)*	68	0.58 (0.48, 0.70)**
Cambodia (2014)	536	0.85 (0.71, 1.01)	485	1.14 (0.93, 1.40)	367	1.31 (1.01, 1.70)*
Cameroon (2009)	776	0.84 (0.74, 0.95)**	769	0.88 (0.79, 0.996)*	722	0.89 (0.80, 0.99)*
Colombia (2010)	1482	0.81 (0.46, 1.41)	1482	0.76 (0.44, 1.32)	Non-est^†^	Non-est^†^
Côte d'Ivoire (2007)	552	0.79 (0.70, 0.89)**	520	0.91 (0.80, 1.03)	453	0.88 (0.79, 0.99)*
Ecuador (2012)	2018	0.63 (0.44, 0.89)**	1996	0.74 (0.57, 0.96)*	1996	0.74 (0.57, 0.96)*
Kenya (2007)	Non-est^†^	Non-est^†^	Non-est^†^	Non-est^†^	Non-est^†^	Non-est^†^
Kenya (2010)	609	1.00 (0.68, 1.47)	603	1.01 (0.72, 1.42)	596	1.13 (0.79, 1.60)
Laos (2006)	149	0.59 (0.34, 1.01)	143	0.48 (0.30, 0.77)**	140	0.49 (0.29, 0.82)**
Liberia (2011)	1163	1.00 (0.88, 1.13)	1163	1.06 (0.92, 1.21)	1151	1.04 (0.91, 1.19)
Malawi (2016)	1168	1.12 (0.78, 1.61)	1046	1.19 (0.84, 1.69)	973	1.40 (0.95, 2.05)
Mexico (2006)	6270	0.83 (0.69, 0.996)*	6245	0.89 (0.75, 1.07)	1448	0.82 (0.57, 1.18)
Nicaragua (2005)	1307	0.65 (0.43, 0.97)*	1307	0.89 (0.57, 1.38)	1307	0.88 (0.57, 1.35)
Pakistan (2011)	8687	0.94 (0.87, 1.02)	8604	0.98 (0.89, 1.06)	6075	1.06 (0.94, 1.20)
Papua New Guinea (2005)	755	0.75 (0.57, 0.99)*	755	0.76 (0.57, 1.03)	716	0.80 (0.58, 1.09)
Philippines (2011)	1726	0.63 (0.47, 0.85)**	1674	0.65 (0.49, 0.86)**	1657	0.65 (0.48, 0.88)**
Rwanda (2010)	574	0.71 (0.27, 1.86)	571	0.64 (0.25, 1.64)	571	0.59 (0.24, 1.43)

1 Values are prevalence ratios (PRs) (95% CIs). Unimproved sanitation is the reference group. Anemia was defined as having an altitude-adjusted hemoglobin concentration <110 g/L, except for Bangladesh (2010 and 2012), Cambodia, Nicaragua, Pakistan, Philippines, Burkina Faso, Cameroon, Côte d'Ivoire, Kenya (2007 and 2010), and Liberia, where altitude was not available. We used Poisson regressions with log link function and robust variance estimation to calculate associations (PR; 95% CI) between anemia and household water source or sanitation; ^*,**^significant association: *P < 0.05, **P < 0.01. AGP, α-1-acid glycoprotein; BRINDA, Biomarkers Reflecting Inflammation and Nutritional Determinants of Anemia; CRP, C-reactive protein; N/A, not available; SES, socioeconomic status. ^†^Nonestimable due to zero-cell issue.

2 Adjusted for child sex, child age in months (continuous), household SES, maternal education (no education, primary education, secondary education, or university), and/or type of residence (rural/urban), when available. SES was a 3-level ordinal variable created from asset quintiles or country income variables. Specifically, the first and second quintiles/categories were collapsed as “low SES,” the third and fourth quintiles/categories were collapsed as “medium SES,” and the fifth quintile/category was converted as “high SES.”

3 Adjusted for child sex, child age in months (continuous), household SES, maternal education (no education, primary education, secondary education, or university), and/or type of residence (rural/urban), when available. Further adjusted for inflammation (yes/no). Inflammation was defined as having a CRP concentration >5 mg/L or an AGP concentration >1 g/L.

**FIGURE 2:**
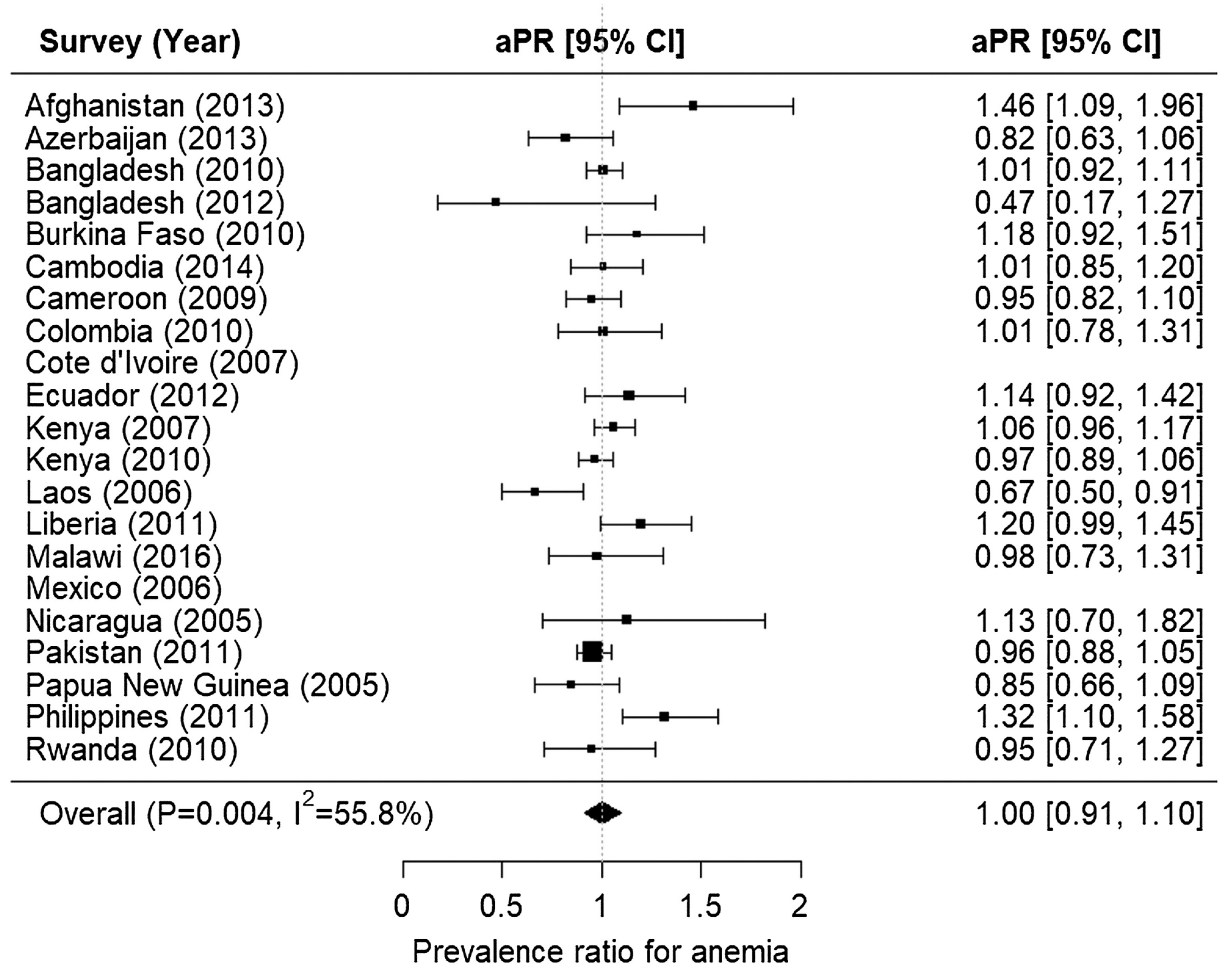
Forest plot for associations between anemia among preschool children and improved household water source: the Biomarkers Reflecting Inflammation and Nutritional Determinants of Anemia (BRINDA) project. Adjusted prevalence ratio (aPR) adjusted for child sex, child age in months (continuous), household socioeconomic status (SES), maternal education (no education, primary education, secondary education, or university), and/or type of residence (rural/urban). Anemia was defined as having an altitude-adjusted hemoglobin concentration <110 g/L, except for Bangladesh (2010 and 2012), Cambodia, Nicaragua, Pakistan, Philippines, Burkina Faso, Cameroon, Côte d'Ivoire, Kenya (2007 and 2010), and Liberia, where altitude was not available. Water was defined as improved or unimproved household water source. SES was a 3-level ordinal variable created from asset quintiles or country income variables. Specifically, the first and second quintiles/categories were collapsed as “low SES,” the third and fourth quintiles/categories were collapsed as “medium SES,” and the fifth quintile/category was converted as “high SES.”

**FIGURE 3:**
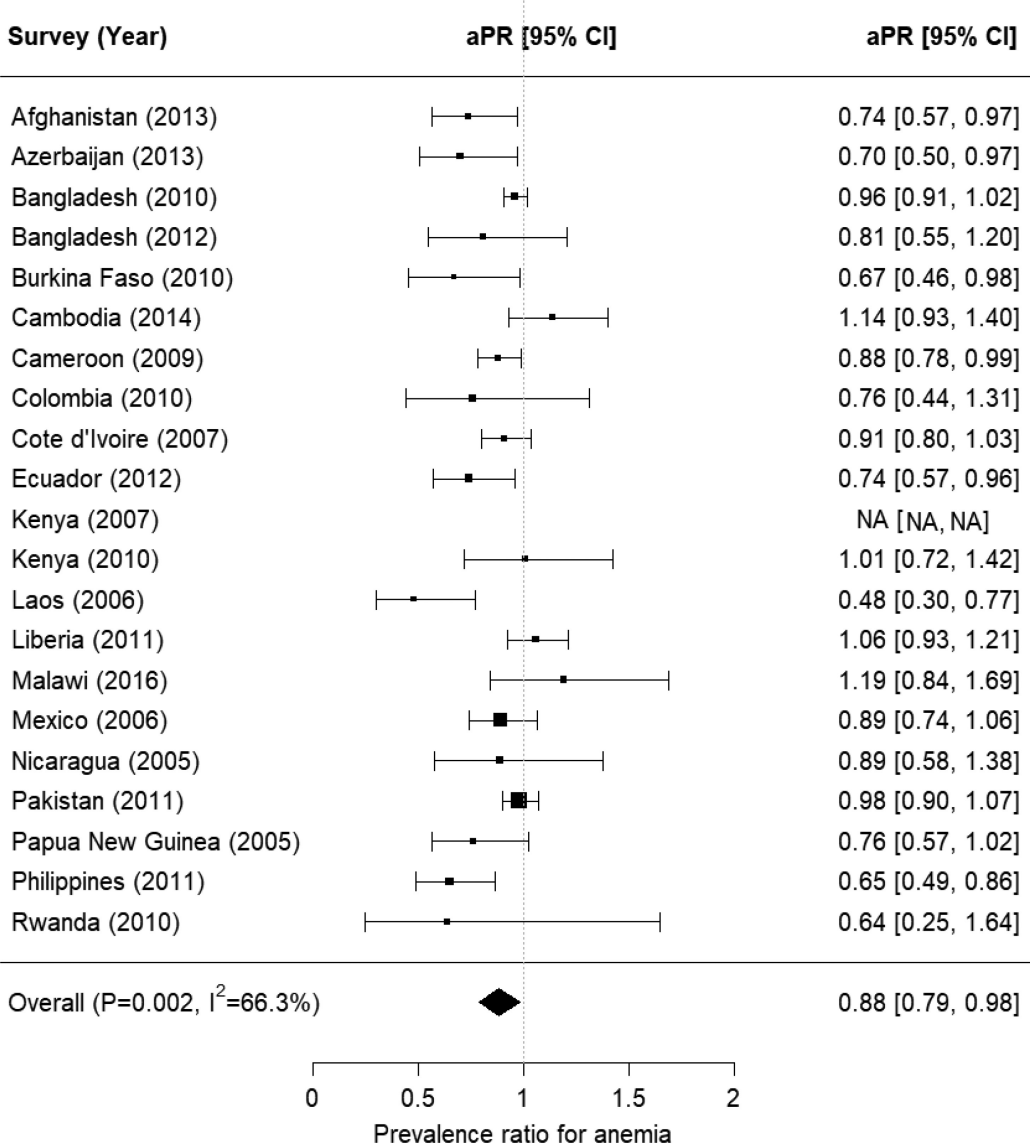
Forest plot for associations between anemia among preschool children and improved household sanitation: the Biomarkers Reflecting Inflammation and Nutritional Determinants of Anemia (BRINDA) project. Adjusted prevalence ratio (aPR) adjusted for child sex, child age in months (continuous), household socioeconomic status (SES), maternal education (no education, primary education, secondary education, or university), and/or type of residence (rural/urban). Anemia was defined as having an altitude-adjusted hemoglobin concentration <110 g/L, except for Bangladesh (2010 and 2012), Cambodia, Nicaragua, Pakistan, Philippines, Burkina Faso, Cameroon, Côte d'Ivoire, Kenya (2007 and 2010), and Liberia, where altitude was not available. Sanitation was defined as improved or unimproved household sanitation, and unimproved sanitation included unimproved sanitation and open defecation. SES was a 3-level ordinal variable created from asset quintiles or country income variables. Specifically, the first and second quintiles/categories were collapsed as “low SES,” the third and fourth quintiles/categories were collapsed as “medium SES,” and the fifth quintile/category was converted as “high SES.”

The meta-analysis showed no association between improved household water and anemia, after adjusting for child sex, child age in months, household SES, maternal education, and/or type of residence (aPR = 1.00; 95% CI: 0.91, 1.10) (**Figure 2**). Notably, there was some evidence of heterogeneity in relations between anemia and improved household water source across surveys (*P *< 0.01; *I*^2^ = 55.8%) (Figure 2). However, the meta-analysis showed a protective association between improved household sanitation and anemia, after adjusting for child sex, child age in months, household SES, maternal education, and/or type of residence (aPR = 0.88; 95% CI: 0.79, 0.98) (**Figure 3**). There was substantial heterogeneity in relations between anemia and improved household sanitation across surveys (*P *< 0.01; *I*^2^ = 66.3%) (Figure 3).

## Discussion

We used population-based data of 35,963 PSC from 21 surveys to describe associations between anemia and household water source or sanitation. Notably, the percentages of participants with access to an improved household water source or sanitation varied greatly across countries, as did the prevalence of inflammation and anemia. Few surveys showed any associations between anemia and an improved household water source.

However, 7 of 20 surveys showed a significant association between anemia and unimproved household sanitation, controlling for child sex, age (months), household SES, maternal education, and/or type of residence. These results indicate that poor household sanitation can contribute to anemia in some settings. However, in the majority of surveys assessed, the relations between sanitation and anemia were nonsignificant. There was also high heterogeneity (*I*^2^ >55%) across surveys. Further, the results did not suggest a mediating effect of inflammation on the relations between anemia and household sanitation across surveys except for Burkina Faso, Cambodia, and Côte d'Ivoire. More sensitive indicators of enteric dysfunction, such as urinary lactulose to mannitol (L:M) ratio (29), might be needed to better understand the mechanisms of inflammation-mediated anemia.

Other observational studies have also found mixed results when exploring the relation between WASH components and anemia. Unimproved sanitation was associated with higher odds of anemia in PSC based on pooled survey data from Bangladesh, Laos, Pakistan, and the Philippines (OR = 1.67; 95% CI: 1.21, 2.30) (6). Another pooled analysis of 60,416 PSC from 27 sub-Saharan African countries participating in the Demographic and Health Surveys (DHS) between 2008 and 2014 showed that unimproved toilet was associated with lower mean Hb values ($\beta $ = −0.047; 95% CI: −0.086, −0.007) (17). Baseline data from the Global Scaling Up Rural Sanitation project showed that the proportion of anemic children was slightly lower for households with improved sanitation (68.7% compared with 72.8%) or with improved water source (70.5% compared with 73.0%) in Indonesia (14). In contrast, a study reported that a lack of sanitation access at the community level was a significant risk factor for anemia independent of sanitation access at the household level (19). The hypothesized mechanism behind this was that community-level sanitation might act through a type of “herd-immunity” (30), especially against enteric diseases.

A recent analysis of DHS data utilized cross-sectional health survey data and meta-analysis techniques similar to those used here to explore the associations between water, sanitation, and anemia (31). The authors found PSC exposed to unimproved sanitation facilities had higher odds of anemia compared to those who had improved sanitation facilities in 18 of 45 countries, adjusting for sex and age (31). Notably, the authors only conducted multivariable logistic regressions in 3 countries that showed strong unadjusted ORs and represented different parts of the world (31). The 3 countries they selected were India, Burundi, and Senegal, where they adjusted for age, sex, residence, education, wealth, iron supplementation, and deworming. In alignment with our findings, they found all 3 countries showed an association between unimproved and shared sanitation facilities and higher odds of anemia, although 1 of the associations was nonsignificant (31). Additionally, they also found a mix of associations between water source and anemia (31).

Evidence from randomized trials on the effect of improved WASH on anemia in children has been mixed, but most studies have reported null findings. A substudy nested in the WASH Benefits cluster-randomized trial in rural Kenya enrolled households with a pregnant woman who was in her second or third trimester between November 2012 and May 2014, whereas its companion study enrolled households in rural Bangladesh between May 2012 and July 2013 (13). In Kenya, the authors found a 3.1%, 12.6%, and 21.5% lower anemia prevalence in the WASH, Nutrition, and WASH + Nutrition groups compared with the control group, respectively (13). Furthermore, there was an 8.9% lower anemia prevalence in the WASH + Nutrition group compared with the Nutrition group, although this difference was nonsignificant (13). In Bangladesh, there was a 4.6%, 8.7%, and 9.5% lower anemia prevalence in the WASH, Nutrition, and WASH + Nutrition groups compared with the control group, respectively (13). However, there was no added value of WASH + Nutrition over Nutrition alone (13). Notably, the overall anemia prevalence in Kenya was much higher than that in Bangladesh, which might explain the greater benefits of the interventions in Kenya (13). On the other hand, the Sanitation Hygiene Infant Nutrition Efficacy (SHINE) study in rural Zimbabwe reported that a WASH intervention (construction of a ventilated improved pit latrine, provision of 2 handwashing stations, liquid soap, chlorine, and play space plus hygiene counseling) had no effect on the mean Hb concentration among children at age 18 mo born to mothers enrolled between November 2012 and March 2015 (15).

These contradictory results, together with the results we found in our analysis, indicate that the potential link between childhood anemia and household water source or sanitation can differ in various settings, as evidenced by the heterogeneity in relations between anemia and improved household water source or sanitation across surveys. Different contexts have different infectious disease pathogens and burdens, dietary practices, accessibility to health services, and other factors that can modify the relation between anemia and household water source or sanitation.

A strength of this analysis is the large sample size and the household-level data on drinking water source and sanitation. We relied on data from a large number of surveys that included biomarkers of inflammation from a wide range of geographical regions, and included a broad age range of PSC. This analysis also explored the distal posited pathway between an “unhealthy environment” and anemia (32). Furthermore, we explored the potential mediating effect of inflammation on the association between WASH components and anemia.

There are several caveats to this study. Primarily, we are unable to make causal inference because surveys are cross-sectional. Second, there was not complete information on all WASH variables for all observations. Some surveys assessed water, whereas others only assessed sanitation, and there were so few surveys that included a measure of hygiene that it was impractical to examine across surveys. Notably, there was variation in the sample sizes of the surveys, which would result in different powers in detecting the associations. It is also noteworthy that the home environment is complex, where many other unmeasured factors in the home environment might be associated with childhood anemia, such as solid fuel use (33) or air pollution (34). Data on some important pathogens, such as hookworm and malaria, were also not available in most of our surveys. Additionally, it is important to explore the associations between WASH and micronutrient deficiencies, which was outside the scope of this analysis. Third, due to insufficient sample size within levels of sanitation, we were unable to assess open defecation. More sensitive indicators could be needed to better characterize the associations between WASH and anemia. Finally, the variation in preanalytical factors (35), blood specimen type (36), drop of blood used (37), and measurement techniques (38,39) across surveys might affect Hb readings.

In conclusion, an improved household water source or sanitation was inconsistently associated with anemia in PSC across surveys. Although a negative association between access to improved sanitation and anemia was evident in approximately one-third of surveys, the heterogeneity across surveys is puzzling. Access to an improved water source in general had no association with anemia across surveys. Further research is warranted to explore a more complex conceptual framework of home environment and/or community environment with both structural (e.g., type of toilet and water source) and behavioral components (e.g., handwashing and infant feces disposal) on the risk of childhood anemia.

## Supplementary Material

nqaa148_Supplemental_File

## Notes

Supported by the Bill & Melinda Gates Foundation, Centers for Disease Control and Prevention, Eunice Kennedy Shriver National Institute of Child Health and Human Development, HarvestPlus, and the United States Agency for International Development.

Publication costs for this supplement were defrayed in part by the payment of page charges. The opinions expressed in this publication are those of the authors and are not attributable to the sponsors or the publisher, Editor, or Editorial Board of *The American Journal of Clinical Nutrition*.

Supplemental Tables 1 and 2 are available from the “Supplementary data” link in the online posting of the article and from the same link in the online table of contents at https://academic.oup.com/ajcn/.

Abbreviations used: AGP, α-1-acid glycoprotein; aPR, adjusted prevalence ratio; BRINDA, Biomarkers Reflecting Inflammation and Nutritional Determinants of Anemia; cPR, crude prevalence ratio; CRP, C-reactive protein; DHS, Demographic and Health Surveys; Hb, hemoglobin; PR, prevalence ratio; PSC, preschool children; SES, socioeconomic status; WASH, water, sanitation, and hygiene.
