# Supplementary material for: Association between anemia and household water source or sanitation in preschool children: the Biomarkers Reflecting Inflammation and Nutritional Determinants of Anemia (BRINDA) project
Source: Am J Clin Nutr. 2020 Aug 4;112(Suppl 1):488S–497S. doi: 10.1093/ajcn/nqaa148 (PMC7396266; doi:10.1093/ajcn/nqaa148)
Supplement: nqaa148_Supplemental_File [file nqaa148_supplemental_file.docx]

Title: Adjusting iron and vitamin A status in settings of inflammation: a sensitivity analysis of the Biomarkers Reflecting Inflammation and Nutritional Determinants of Anemia (BRINDA) approach

Authors: Sorrel ML Namaste, Jiangda Ou, Anne M Williams, Melissa F. Young, Emma X Yu, Parminder S Suchdev

**Supplementary Table 1.** **Biomarker laboratory methods for the biomarkers included in the analyses from the surveys in the BRINDA project database^1^**

| **Country (Year)** | **CRP** | **AGP** | **Ferritin** | **sTfR** | **Retinol** | **RBP** |
| --- | --- | --- | --- | --- | --- | --- |
| Afghanistan (2013) | Immunoassay | Turbidmetry | Not measured | Not measured | HPLC | Not measured |
| Azerbaijan (2013) | Sandwich ELISA | Sandwich ELISA | Sandwich ELISA | Sandwich ELISA | Not measured | Sandwich ELISA |
| Bangladesh (2008) | Immuno-turbidmetry | Immuno-turbidmetry | Not measured | Not measured | Not measured | Not measured |
| Bangladesh (2010) | Sandwich ELISA | Sandwich ELISA | Sandwich ELISA | Sandwich ELISA | Not measured | Sandwich ELISA |
| Bangladesh (2012) | Sandwich ELISA | Sandwich ELISA | Sandwich ELISA | Not measured | HPLC | Not measured |
| Burkina Faso (2010) | Turbidmetry | Turbidmetry | Turbidimetry | Sandwich ELISA | HPLC^†^ | Sandwich ELISA |
| Cambodia (2014) | Sandwich ELISA | Sandwich ELISA | Sandwich ELISA | Sandwich ELISA | Not measured | Sandwich ELISA |
| Cameroon (2009) | Sandwich ELISA | Sandwich ELISA | Sandwich ELISA | Sandwich ELISA | HPLC^†^ | Sandwich ELISA |
| Colombia (2010) | Turbidimetry | Not measured | CLIA^†^ | Not measured | HPLC^†^ | Not measured |
| Cote d'Ivoire (2007) | Sandwich ELISA | Sandwich ELISA | Sandwich ELISA | Sandwich ELISA | Not measured | Sandwich ELISA |
| Ecuador (2012) | Nephelometry | Not measured | CLIA^†^ | Not measured | HPLC^†^ | Not measured |
| Georgia (2009) | Turbidimetry | Not measured | Turbidimetry^†^ | Not measured | Not measured | Not measured |
| India (2011) | Immuno-turbidimetry | Immuno-turbidimetry | Immuno-turbidimetry | Immuno-turbidimetry | Not measured | Not measured |
| Kenya (2007) | Sandwich ELISA | Sandwich ELISA | Sandwich ELISA | Sandwich ELISA | Not measured | Sandwich ELISA |
| Kenya (2010) | Sandwich ELISA | Sandwich ELISA | Sandwich ELISA | Sandwich ELISA | Not measured | Sandwich ELISA |
| Laos (2006) | Sandwich ELISA | Sandwich ELISA | Sandwich ELISA | Sandwich ELISA | Not measured | Not measured |
| Liberia (2011) | Sandwich ELISA | Sandwich ELISA | Sandwich ELISA | Sandwich ELISA | Not measured | Sandwich ELISA |
| Malawi (2016) | Sandwich ELISA | Sandwich ELISA | Sandwich ELISA | Sandwich ELISA | HPLC^†^ | Sandwich ELISA |
| Mexico (2006) | Nephelometry | Not measured | Immunoassay^†^ | Immunoassay^†^ | Not measured | Not measured |
| Mexico (2012) | Nephelometry | Not measured | CLIA^†^ | Not measured | HPLC^†^ | Not measured |
| Mongolia (2006) | Not measured | Immuno-turbidmetry | MEIA^†^ | Not measured | HPLC^†^ | Not measured |
| Nicaragua (2005) | Not measured | Turbidmetry | Immunoassay^†^ | Not measured | HPLC^†^ | Not measured |
| Nigeria (2012) | Immunoassay^†^ | ELISA | Sandwich ELISA | Sandwich ELISA | HPLC^†^ | Sandwich ELISA |
| Pakistan (2011)^2^ | Immunoassay | Turbidimetry | Turbidimetry | Not measured | HPLC | Not measured |
| Philippines (2011) | Sandwich ELISA | Sandwich ELISA | Sandwich ELISA | Sandwich ELISA | Not measured | Sandwich ELISA |
| PNG (2005) | Sandwich ELISA | Sandwich ELISA | Not measured | Sandwich ELISA | Not measured | Sandwich ELISA |
| Rwanda (2010) | Sandwich ELISA | Sandwich ELISA | Sandwich ELISA | Sandwich ELISA | Not measured | Sandwich ELISA |
| United Kingdom (2014) | Siemens/DADE automated analyzer | Not measured | Immuno-ephelometry^†^ | Unknown^†^ | HPLC^†^ | Not measured |
| United States (2003-06) | Nephelometry | Not measured | Immuno-radiometry^†^ | Immuno-turbidimetry^†^ | Not measured | Not measured |
| Vietnam (2010) | ELISA | Not measured | ELISA^†^ | Not measured | HPLC^†^ | Not measured |
| Zambia (2009) | Radial Immunodiffusion | Radial Immunodiffusion | ELISA | Not measured | HPLC | Not measured |

^1^The VitMin Laboratory analyzed all samples in which the sandwich ELISA technique was used.

^2^Pakistan 2011 is included in BRINDA phase 1 for children but included in BRINDA phase 2 for women. Only AGP data was available in BRINDA phase 1, and CRP data became available in BRINDA phase 2 for women.

^†^Not presented in this analysis.

AGP, alpha-1-acid glycoprotein; BRINDA, Biomarkers Reflecting Inflammation and Nutritional Determinants of Anemia; CLIA, chemiluminescent immunoassay; CRP, C-Reactive Protein; HPLC, high performance liquid chromatography; MEIA, microparticle enzyme immunoassay; PNG, Papua New Guinea; RBP, retinol binding protein; sTfR, soluble transferrin receptor.

**Supplementary Table 2. Kendall’s tau coefﬁcient matrix relating AGP, CRP, ferritin, sTfR, RBP or retinol, among preschool children, BRINDA project^1^**

| **Country** | **AGP** |  | **Ferritin** | |  | **sTfR** | |  | **Retinol or RBP** | |
| --- | --- | --- | --- | --- | --- | --- | --- | --- | --- | --- |
|  | **CRP** |  | **CRP** | **AGP** |  | **CRP** | **AGP** |  | **CRP** | **AGP** |
| **BRINDA phase 1** |  |  |  |  |  |  |  |  |  |  |
| Bangladesh 2010 | 0.49 |  | 0.18 | 0.19 |  | 0.00 | 0.09 |  | -0.26 | -0.18 |
| Cameroon 2009 | 0.56 |  | 0.35 | 0.32 |  | 0.17 | 0.18 |  | -0.30 | -0.23 |
| Côte d’Ivoire 2007 | 0.52 |  | 0.36 | 0.27 |  | 0.16 | 0.28 |  | -0.32 | -0.27 |
| Kenya 2007 | 0.51 |  | 0.31 | 0.30 |  | 0.04 | 0.08 |  | -0.31 | -0.27 |
| Kenya 2010 | 0.63 |  | 0.44 | 0.44 |  | 0.17 | 0.24 |  | -0.37 | -0.32 |
| Laos 2006 | 0.48 |  | 0.13 | 0.15 |  | 0.09 | 0.08 |  | NA | NA |
| Liberia 2011 | 0.50 |  | 0.33 | 0.30 |  | 0.07 | 0.15 |  | -0.26 | -0.23 |
| PNG 2005 | 0.54 |  | NA | NA |  | 0.20 | 0.20 |  | -0.33 | -0.19 |
| Philippines 2011 | 0.61 |  | 0.16 | 0.19 |  | 0.01 | 0.08 |  | -0.35 | -0.25 |
| **BRINDA phase 2** |  |  |  |  |  |  |  |  |  |  |
| Afghanistan 2013 | 0.46 |  | 0.06 | 0.05 |  | NA | NA |  | -0.20 | -0.2 |
| Azerbaijan 2013 | 0.53 |  | 0.25 | 0.33 |  | -0.05 | 0.00 |  | -0.26 | -0.18 |
| Bangladesh 2012 | 0.54 |  | 0.19 | 0.22 |  | NA | NA |  | -0.10 | -0.10 |
| Burkina Faso 2010 | 0.41 |  | 0.32 | 0.36 |  | 0.11 | 0.44 |  | -0.25 | 0.11 |
| Cambodia 2014 | 0.43 |  | 0.19 | 0.39 |  | 0.17 | 0.54 |  | 0.08 | 0.48 |
| Malawi 2016 | 0.46 |  | 0.21 | 0.22 |  | 0.16 | 0.19 |  | -0.28 | -0.17 |
| Nigeria 2012 | 0.58 |  | 0.43 | 0.40 |  | 0.21 | 0.28 |  | -0.25 | -0.12 |
| Rwanda 2010 | 0.42 |  | 0.05 | 0.15 |  | -0.01 | 0.09 |  | -0.26 | -0.21 |
| Zambia 2009 | 0.25 |  | 0.26 | 0.34 |  | NA | NA |  | -0.10 | -0.20 |
| ^1^AGP, alpha-1-acid glycoprotein; BRINDA, Biomarkers Reflecting Inflammation and Nutritional Determinants of Anemia; CRP, C-Reactive Protein; NA, not available; PNG, Papua New Guinea; RBP, retinol binding protein; sTfR, soluble transferrin receptor. | | | | | | | | | | |

**Supplementary Table 3.** **Kendall's tau coefficient matrix relating AGP, CRP, ferritin, sTfR, RBP or retinol, among women of reproductive age, BRINDA project^1^**

| **Country** | **AGP** |  | **Ferritin** | |  | **sTfR** | |  | **Retinol or RBP** | |
| --- | --- | --- | --- | --- | --- | --- | --- | --- | --- | --- |
|  | **CRP** |  | **CRP** | **AGP** |  | **CRP** | **AGP** |  | **CRP** | **AGP** |
| **BRINDA phase 1** | |  |  |  |  |  |  |  |  |  |
| PNG 2005 | 0.38 |  | NA | NA |  | 0.09 | 0.17 |  | -0.13 | -0.01 |
| Cameroon 2009 | 0.41 |  | 0.18 | 0.16 |  | 0.07 | 0.17 |  | -0.10 | -0.04 |
| Côte d’Ivoire 2007 | 0.39 |  | 0.15 | 0.15 |  | 0.06 | 0.17 |  | -0.02 | 0.00 |
| Laos 2006 | 0.26 |  | 0.16 | 0.06 |  | -0.04 | 0.15 |  | NA | NA |
| Liberia 2011 | 0.38 |  | 0.17 | 0.16 |  | 0.02 | 0.12 |  | -0.08 | 0.04 |
| **BRINDA phase 2** | |  |  |  |  |  |  |  |  |  |
| Afghanistan 2013 | 0.29 |  | 0.09 | 0.19 |  | NA | NA |  | 0.00 | 0.00 |
| Azerbaijan 2013 | 0.49 |  | 0.17 | 0.14 |  | -0.03 | 0.06 |  | 0.11 | 0.14 |
| Bangladesh 2012 | 0.37 |  | 0.12 | 0.04 |  | NA | NA |  | 0.00 | 0.00 |
| Burkina Faso 2010 | 0.24 |  | 0.17 | 0.20 |  | 0.03 | 0.34 |  | 0.04 | 0.31 |
| Cambodia 2014 | 0.26 |  | 0.18 | 0.32 |  | 0.13 | 0.63 |  | 0.12 | 0.50 |
| India 2011 | 0.49 |  | 0.19 | 0.15 |  | -0.05 | 0.04 |  | NA | NA |
| Malawi 2016 | 0.34 |  | 0.05 | 0.06 |  | 0.07 | 0.16 |  | -0.06 | 0.01 |
| Nigeria 2012 | 0.36 |  | 0.12 | 0.19 |  | 0.05 | 0.17 |  | -0.10 | 0.07 |
| Pakistan 2011 | 0.11 |  | 0.04 | 0.05 |  | 0.04 | 0.05 |  | 0.00 | 0.00 |
| Rwanda 2010 | 0.28 |  | -0.02 | 0.10 |  | -0.01 | 0.10 |  | -0.17 | -0.07 |

^1^AGP, alpha-1-acid glycoprotein; BRINDA, Biomarkers Reflecting Inflammation and Nutritional Determinants of Anemia; CRP, C-Reactive Protein; NA, not available; RBP, retinol binding protein; sTfR, soluble transferrin receptor.

| **Supplementary Table 4. Estimated prevalence of unadjusted depleted iron stores and iron-deficient erythropoiesis among children 6-59 months, BRINDA Project^1^** | | | | |
| --- | --- | --- | --- | --- |
|  | ***n*** | **Ferritin (< 12 µg/L)** | ***n*** | **sTfR (> 8.3 mg/L)** |
| **BRINDA phase 1** |  |  |  |  |
| Bangladesh 2010 | 1493 | 8.0 [5.9,10.7] | 1493 | 55.7 [52.0,59.4] |
| Cameroon 2009 | 792 | 14.8 [12.3,17.8] | 792 | 68.5 [63.9,72.8] |
| Côte d’Ivoire 2007 | 746 | 11.7 [9.2,14.8] | 746 | 23.9 [20.1,28.1] |
| Kenya 2007 | 896 | 38.7 [34.7,42.9] | 896 | 35.5 [31.2,40.0] |
| Kenya 2010 | 849 | 19.1 [15.9,22.7] | 849 | 74.8 [70.9,78.3] |
| Laos 2006 | 481 | 16.6 [12.8,21.2] | 481 | 4.1 [2.4,6.9] |
| Liberia 2011 | 1434 | 20.4 [18.0,23.2] | 1434 | 76.7 [73.3,79.9] |
| Philippines 2011 | 1767 | 26.2 [23.1,29.5] | 1767 | 13.5 [10.5,17.2] |
| PNG 2005 | NA |  | 868 | 25.1 [20.9,29.8] |
| **BRINDA phase 2** |  |  |  |  |
| Afghanistan | 665 | 22.0 [17.9,26.6] | NA |  |
| Azerbaijan 2013 | 1053 | 13.9 [11.5,16.7] | 1053 | 7.9 [6.1,10.1] |
| Bangladesh 2012 | 467 | 9.8 [5.9,15.7] | NA |  |
| Burkina Faso 2010 | 125 | 0 [0, 0] | 125 | 85.8 [77.4,91.4] |
| Cambodia 2014 | 665 | 3.8 [2.4,5.9] | 665 | 49.3 [44.2,54.5] |
| Malawi 2016 | 1102 | 10.7 [7.8,14.6] | 1102 | 55.2 [49.0,61.3] |
| Nigeria 2011-12 | 547 | 5.1 [3.4,7.7] | 547 | 59.8 [53.8,65.5] |
| Rwanda 2010 | 576 | 5.3 [3.5,7.8] | 576 | 3.1 [1.8,5.2] |
| Zambia 2009 | 410 | 5.4 [2.7,10.2] | NA |  |
| ^1^Values are percent [95% CI]; BRINDA, Biomarkers for Inflammation and Nutritional Determinants of Anemia; NA, not available; PNG, Papua New Guinea; sTfR, soluble transferrin receptor. | | | | |
|  |  |  |  |  |
|  |  |  |  |  |

| **Supplementary Table 5. Estimated prevalence of unadjusted depleted iron stores and iron-deficient erythropoiesis among women of reproductive age 15-49 yr, BRINDA Project^1^** | | | | |
| --- | --- | --- | --- | --- |
|  | ***n*** | **Ferritin (< 15 µg/L)** | ***n*** | **sTfR (> 8.3 mg/L)** |
| BRINDA Phase 1 |  |  |  |  |
| Cameroon 2009 | 760 | 12.8 [10.4,15.7] | 760 | 33.8 [29.2,38.7] |
| Côte d’Ivoire 2007 | 834 | 13.4 [10.8,16.5] | 834 | 31.1 [27.2,35.4] |
| Laos 2006 | 816 | 22.7 [17.9,28.3] | 816 | 5.8 [3.8,8.6] |
| Liberia 2011 | 1942 | 17.9 [15.6,20.4] | 1942 | 28.3 [25.3,31.5] |
| PNG 2005 | NA |  | 746 | 17.7 [14.4,21.6] |
| BRINDA Phase 2 |  |  |  |  |
| Afghanistan 2013 | 1050 | 25.9 [21.1,31.3] | NA |  |
| Azerbaijan 2013 | 2656 | 30.7 [28.5,33.1] | 2656 | 18.5 [16.7,20.4] |
| Bangladesh 2012 | 876 | 7.6 [5.2,10.9] | NA |  |
| Burkina Faso 2010 | 129 | 3.1 [1.1,8.2] | 129 | 80.8 [65.2,90.4] |
| Cambodia 2014 | 705 | 2.7 [1.5,4.6] | 705 | 33.5 [26.5,41.3] |
| Malawi 2016 | 776 | 11.4 [8.5,15.1] | 776 | 24.5 [20.9,28.6] |
| Nigeria 2011-12 | 620 | 8.7 [6.2,12.2] | 620 | 36.1 [32.3,40.1] |
| Pakistan 2011 | 5988 | 35.6 [34.1,37.1] | 4968 | 16.5 [15.2,17.9] |
| Rwanda 2010 | 596 | 3.9 [2.5,6.1] | 596 | 3.0 [1.8,4.8] |
| Gujarat, India 2011 | 323 | 45.8 [38.4,53.4] | 325 | 38.8 [32.7,45.2] |
| ^1^Values are percent [95% CI]; BRINDA, Biomarkers for Inflammation and Nutritional Determinants of Anemia; NA, not available; PNG, Papua New Guinea; sTfR, soluble transferrin receptor. | | | | |
|  |  |  |  |  |
|  |  |  |  |  |

| **Supplementary Table 6. Estimated prevalence of unadjusted vitamin A deficiency among children 6-59 months, BRINDA Project^1^** | | |
| --- | --- | --- |
|  | ***n*** | **RBP or retinol (< 0.70 µmol/L)** |
| **BRINDA phase 1** |  |  |
| Bangladesh 2010 | 1493 | 17.0 [14.5,19.8] |
| Cameroon 2009 | 792 | 28.7 [24.6,33.1] |
| Côte d’Ivoire 2007 | 746 | 24.0 [20.4,27.9] |
| Kenya 2007 | 896 | 23.0 [19.4,27.1] |
| Kenya 2010 | 849 | 29.6 [25.9,33.5] |
| Liberia 2011 | 1434 | 24.7 [21.4,28.3] |
| Philippines 2011 | 1767 | 6.9 [5.3,8.9] |
| PNG 2005 | 868 | 24.7 [20.7,29.2] |
| **BRINDA phase 2** |  |  |
| Afghanistan | 657 | 47.6 [42.2,53.1] |
| Azerbaijan 2013 | 1053 | 12.2 [9.3,15.8] |
| Bangladesh 2012 | 458 | 20.6 [14.9,27.9] |
| Burkina Faso 2010 | 125 | 8.9 [4.5,17.0] |
| Cambodia 2014 | 665 | 9.9 [7.0,13.8] |
| Malawi 2016 | 1102 | 24.1 [20.2,28.5] |
| Nigeria 2011-12 | 547 | 25.6 [20.9,30.9] |
| Rwanda 2010 | 576 | 10.7 [8.1,14.0] |
| Zambia 2009 | 389 | 56.6 [49.4,63.4] |
| ^1^Values are percent [95% CI]; BRINDA, Biomarkers for Inflammation and Nutritional Determinants of Anemia; NA, not available; PNG, Papua New Guinea; RBP, retinol binding protein. | | |
|  |  |  |
|  |  |  |

| **Supplementary Table 7. Estimated prevalence of adjusted depleted iron stores and iron-deficient erythropoiesis among children 6-59 months, BRINDA Project^1^** | | | | |
| --- | --- | --- | --- | --- |
|  | ***n*** | **Ferritin (< 12 µg/L)** | ***n*** | **sTfR (> 8.3 mg/L)** |
| **BRINDA phase 1** |  |  |  |  |
| Bangladesh 2010 | 1493 | 15.9 [13.5,18.7] | 1493 | 47.1 [43.9,50.3] |
| Cameroon 2009 | 792 | 34.9 [30.8,39.2] | 792 | 44.7 [40.3,49.1] |
| Côte d’Ivoire 2007 | 746 | 39.5 [35.5,43.6] | 746 | 8.4 [6.4,11.1] |
| Kenya 2007 | 896 | 72.4 [68.6,76.0] | 896 | 27.3 [23.8,31.2] |
| Kenya 2010 | 849 | 53.4 [49.6,57.1] | 849 | 51.9 [47.8,56.1] |
| Laos 2006 | 481 | 26.4 [21.9,31.4] | 481 | 3.3 [1.8,6.1] |
| Liberia 2011 | 1434 | 55.6 [51.5,59.6] | 1434 | 55.9 [52.2,59.5] |
| Philippines 2011 | 1767 | 34.8 [31.4,38.3] | 1767 | 12.1 [9.5,15.2] |
| PNG 2005 | NA |  | 868 | 10.5 [7.4,14.6] |
| **BRINDA phase 2** |  |  |  |  |
| Afghanistan | 665 | 24.2 [20.1,28.8] | NA |  |
| Azerbaijan 2013 | 1053 | 22.4 [18.9,26.2] | 1053 | 6.7 [5.3,8.5] |
| Bangladesh 2012 | 467 | 13.6 [9.1,19.9] | NA |  |
| Burkina Faso 2010 | 125 | 8.7 [3.5,19.9] | 125 | 15.5 [8.4,27.0] |
| Cambodia 2014 | 665 | 4.9 [3.4,7.1] | 665 | 29.8 [26.5,33.4] |
| Malawi 2016 | 1102 | 21.9 [17.0,27.7] | 1102 | 42.4 [36.7,48.4] |
| Nigeria 2011-12 | 547 | 18.3 [14.1,23.4] | 547 | 30.0 [24.7,35.9] |
| Rwanda 2010 | 576 | 6.2 [4.3,8.9] | 576 | 2.5 [1.5,4.2] |
| Zambia 2009 | 410 | 16.6 [11.5,23.3] | NA |  |
| ^1^Values are percent [95% CI]; Biomarkers for Inflammation and Nutritional Determinants of Anemia; NA, not available; PNG, Papua New Guinea; sTfR, soluble transferrin receptor. | | | | |
|  |  |  |  |  |
|  |  |  |  |  |

| **Supplementary Table 8. Estimated prevalence of adjusted depleted iron stores and iron-deficient erythropoiesis among women of reproductive age 15-49 yr, BRINDA Project^1^** | | | | |
| --- | --- | --- | --- | --- |
|  | ***n*** | **Ferritin (< 15 µg/L)** | ***n*** | **sTfR (> 8.3 mg/L)** |
| **BRINDA Phase 1** |  |  |  |  |
| Cameroon 2009 | 760 | 19.3 [16.1,22.9] | 760 | 20.1 [16.6,24.2] |
| Côte d’Ivoire 2007 | 834 | 22.5 [19.3,26.0] | 834 | 21.4 [18.3,24.8] |
| Laos 2006 | 816 | 26.4 [20.8,32.9] | 816 | 3.9 [2.6,5.8] |
| Liberia 2011 | 1942 | 28.4 [25.3,31.7] | 1942 | 21.9 [19.4,24.7] |
| PNG 2005 | NA |  | 746 | 7.8 [5.8,10.3] |
| **BRINDA Phase 2** |  |  |  |  |
| Afghanistan 2013 | 1050 | 34.0 [28.6,39.7] | NA |  |
| Azerbaijan 2013 | 2656 | 44.0 [41.5,46.5] | 2656 | 16.2 [14.5,17.9] |
| Bangladesh 2012 | 876 | 9.5 [6.8,13.1] | NA |  |
| Burkina Faso 2010 | 129 | 11.1 [5.9,19.8] | 129 | 43.1 [32.5,54.4] |
| Cambodia 2014 | 705 | 3.5 [2.2,5.6] | 705 | 11.2 [8.7,14.4] |
| Malawi 2016 | 776 | 12.7 [9.7,16.3] | 776 | 22.3 [18.6,26.5] |
| Nigeria 2011-12 | 620 | 15.5 [12.2,19.5] | 620 | 26.6 [22.8,30.9] |
| Pakistan 2011 | 5988 | 41.8 [40.1,43.4] | 4968 | 14.2 [13.0,15.6] |
| Rwanda 2010 | 596 | 5.2 [3.4,7.7] | 596 | 1.5 [0.7,3.0] |
| Gujarat, India 2011 | 323 | 57.0 [49.8,63.9] | 325 | 36.0 [30.3,42.1] |
| ^1^Values are percent [95% CI]; BRINDA, Biomarkers for Inflammation and Nutritional Determinants of Anemia; NA, not available; PNG, Papua New Guinea; sTfR, soluble transferrin receptor. | | | | |
|  |  |  |  |  |
|  |  |  |  |  |

| **Supplementary Table 9. Estimated prevalence of adjusted vitamin A deficiency among children 6-59 months, BRINDA Project^1^** | | |
| --- | --- | --- |
|  | ***n*** | **Retinol or RBP (< 0.70 µmol/L)** |
| **BRINDA Phase 1** |  |  |
| Bangladesh 2010 | 1493 | 6.2 [4.5,8.6] |
| Cameroon 2009 | 792 | 9.2 [7.1,11.8] |
| Côte d’Ivoire 2007 | 746 | 3.1 [2.0,4.7] |
| Kenya 2007 | 896 | 6.4 [4.6,8.7] |
| Kenya 2010 | 849 | 8.1 [6.7,9.9] |
| Liberia 2011 | 1434 | 5.4 [4.1,7.1] |
| Philippines 2011 | 1767 | 0.9 [0.5,1.7] |
| PNG 2005 | 868 | 9.8 [7.8,12.3] |
| **BRINDA Phase 2** |  |  |
| Afghanistan | 657 | 37.9 [32.5,43.7] |
| Azerbaijan 2013 | 1053 | 6.2 [4.2,9.0] |
| Bangladesh 2012 | 458 | 14.0 [9.0,21.0] |
| Burkina Faso 2010 | 125 | 7.3 [2.9,17.3] |
| Cambodia 2014 | 665 | 6.5 [4.5,9.5] |
| Malawi 2016 | 1102 | 8.1 [5.8,11.2] |
| Nigeria 2011-12 | 547 | 11.5 [8.5,15.4] |
| Rwanda 2010 | 576 | 4.5 [2.9,6.9] |
| Zambia 2009 | 389 | 43.7 [36.1,51.6] |
| ^1^Values are percent [95% CI]; BRINDA, Biomarkers for Inflammation and Nutritional Determinants of Anemia; NA, not available; PNG, Papua New Guinea; RBP, retinol binding protein. | | |
|  |  |  |
|  |  |  |
